# Carboxyl-terminal modulator protein regulates Akt signaling during skeletal muscle atrophy *in vitro* and a mouse model of amyotrophic lateral sclerosis

**DOI:** 10.1038/s41598-019-40553-2

**Published:** 2019-03-08

**Authors:** Junmei Wang, Colin M. E. Fry, Chandler L. Walker

**Affiliations:** 1 Department of Biomedical and Applied Sciences, Indiana University School of Dentistry, Indianapolis, IN 46202 USA; 2 Department of Anatomy and Cell Biology, Indiana University School of Medicine, Indianapolis, IN 46202 USA; 3 Neuromuscular Research Group, Richard L. Roudebush Veterans Affairs Medical Center, Indianapolis, IN 46202 USA

## Abstract

Amyotrophic lateral sclerosis (ALS) is a progressive neuromuscular disease involving motor neuron death, paralysis and, ultimately, respiratory failure. Motor neuron dysfunction leads to target skeletal muscle atrophy involving dysregulation of downstream cell survival, growth and metabolic signaling. Decreased Akt activity is linked to muscle atrophy in ALS and is associated with increased atrophy gene expression. Unfortunately, the regulating mechanism of Akt activity in atrophic muscle remains unclear. Recent research indicates a role of carboxyl-terminal modulator protein (CTMP) in Akt-signaling related neurologic dysfunction and skeletal muscle metabolism. CTMP is known to bind and reduce Akt phosphorylation and activation. We hypothesized that CTMP expression might progressively increase in ALS skeletal muscle as the disease progresses, downregulating Akt activity. We found that CTMP protein expression significantly increased in hindlimb skeletal muscle in the mSOD1^G93A^ mouse model of ALS in late stages of the disease (*P* < 0.05), which negatively correlated with Akt phosphorylation over this period (*R*^2^ = −0.77). Co-immunoprecipitation of Akt revealed CTMP binding in pre-symptomatic and end-stage skeletal muscle, suggesting a possible direct role in reduced Akt signaling during disease progression. Inflammatory TNFα and downstream cellular degradation process markers for autophagy, lysosome production, and atrophy significantly increased in a pattern corresponding to increased CTMP expression and reduced Akt phosphorylation. In an *in vitro* model of skeletal muscle atrophy, differentiated C2C12 cells exhibited reduced Akt activity and decreased FOXO1 phosphorylation, a process known to promote transcription of atrophy genes in skeletal muscle. These results corresponded with  increased  *Atrogin-1* expression  compared to healthy control cells  (*P* < 0.05). Transfection with CTMP siRNA significantly increased Akt phosphorylation in atrophic C2C12 cells, corresponding to significantly decreased CTMP expression. In conclusion, this is the first study to provide evidence for a link between elevated CTMP expression, downregulated Akt phosphorylation and muscle atrophy in ALS and clearly demonstrates a direct influence of CTMP on Akt phosphorylation in an *in vitro* muscle cell atrophy model.

## Introduction

Motor neuron diseases (MND) are a collection of neurological disorders that affect upper and lower motor neurons of the central nervous system (CNS). The most common form of MND is amyotrophic lateral sclerosis (ALS), which affects individuals at primary productive periods of life and afflicts both upper and lower motor neurons^1–3^. ALS is diagnosed by excluding other possible conditions, and once identified, has typically progressed to advanced stages and prognosis is 3–5 years. No cures for ALS exist and approved therapies have marginal effects on disease progression and survival. A major contributor to the difficulty of identifying and developing effective treatments, aside from delayed diagnosis, is the complex non-cell autonomous nature of the disease. Astrocytes and microglia influence motor neuron survival and health in the CNS, while neuromuscular interactions in the periphery also play a role in disease progression. Targeting multiple cell types and influences of disease onset and progression is difficult, and our understanding of the many factors contributing to ALS remains incomplete.

Much research to date has focused on improving motor neuron survival as a means to improve functional outcomes and slow disease progression; however, due to the dynamic nature of the disease and involvement of various cells and structures, this approach has not proven effective. In the past two decades, our understanding of the pre-symptomatic aspects of disease progression have expanded considerably, and we know that some of the first major outward pre-symptomatic anatomic manifestations of disease is the dismantling of the neuromuscular junction (NMJ) and progressive disconnection of motor neurons from NMJs^4–7^. With this information, further insights into physiologic changes within the skeletal muscle over time in humans and ALS animal models, especially the classic mutant superoxide dismutase 1 (SOD1) mouse model (mSOD1^G93A^)^8^, have been made possible.

As a result of reduced motor stimulation in ALS progression, skeletal muscles atrophy and multiple biochemical processes in skeletal muscle cells have been implicated in this process. Since skeletal muscle is peripheral and directly affected early in ALS progression^5,6,9–11^, assessment of biochemical changes in animal models and human ALS for identification of potential markers of disease has increased in interest. Recent research indicated that a reduction in insulin signaling and expression and activity the serine-threonine kinase, Akt, are associated with progressive muscular atrophy in animal models and correlated with poor survival prognosis in ALS patients^12–14^. Reduced Akt can be correlated with high expression of autophagosome and lysosomal markers through subsequent effects on downstream effectors including the mammalian target of rapamycin (mTOR)^15,16^, and these changes have also been linked to muscle atrophy^17^. Reduced autophagic flux, or increased autophagosome aggregation and lysosome formation have been demonstrated in advanced mSOD1^G93A^ mouse skeletal muscle^17^. Active Akt can also phosphorylate forkhead box (FOXO) proteins, including FOXO1, which prevents them from entering the nucleus and promoting transcription of atrophy genes such as *Atrogin1* (*MAfbx*) and *muscle-ring finger-1* (*MuRF-1*), and thus the expression of these E3-ubiquitin ligases in muscle cells^18,19^. Therefore, downregulation of Akt activity promotes atrophy-associated protein expression and increases muscle degradation processes through such mechanisms.

Akt is activated by phosphorylation at multiple sites through phosphatidylinositol-3-kinase signaling, and binding of carboxyl-terminal modulator protein (CTMP) can prohibit this activation^20^. CTMP is upregulated in neurological disorders such as stroke and traumatic brain injury, and its inhibition increased Akt phosphorylation and signaling and reduced neurological tissue damage^21–23^. Akt activation is linked to myogenesis and atrophy, with its activity increasing and decreasing, respectively. Only recently has the role of CTMP been tied to muscle metabolism, with a reduction in CTMP causing upregulation of Akt phosphorylation and increased myogenesis^24^. Here we report key biochemical changes in the gastrocnemius muscle of the mSOD1^G93A^ amyotrophic mouse model and discuss for the first time, a potential association between CTMP and Akt activation overtime in the muscle of this mouse model.

## Results

### Akt phosphorylation and CTMP expression are inversely correlated, and Akt and CTMP interact in mSOD1^G93A^ muscle

**Figure 1:**
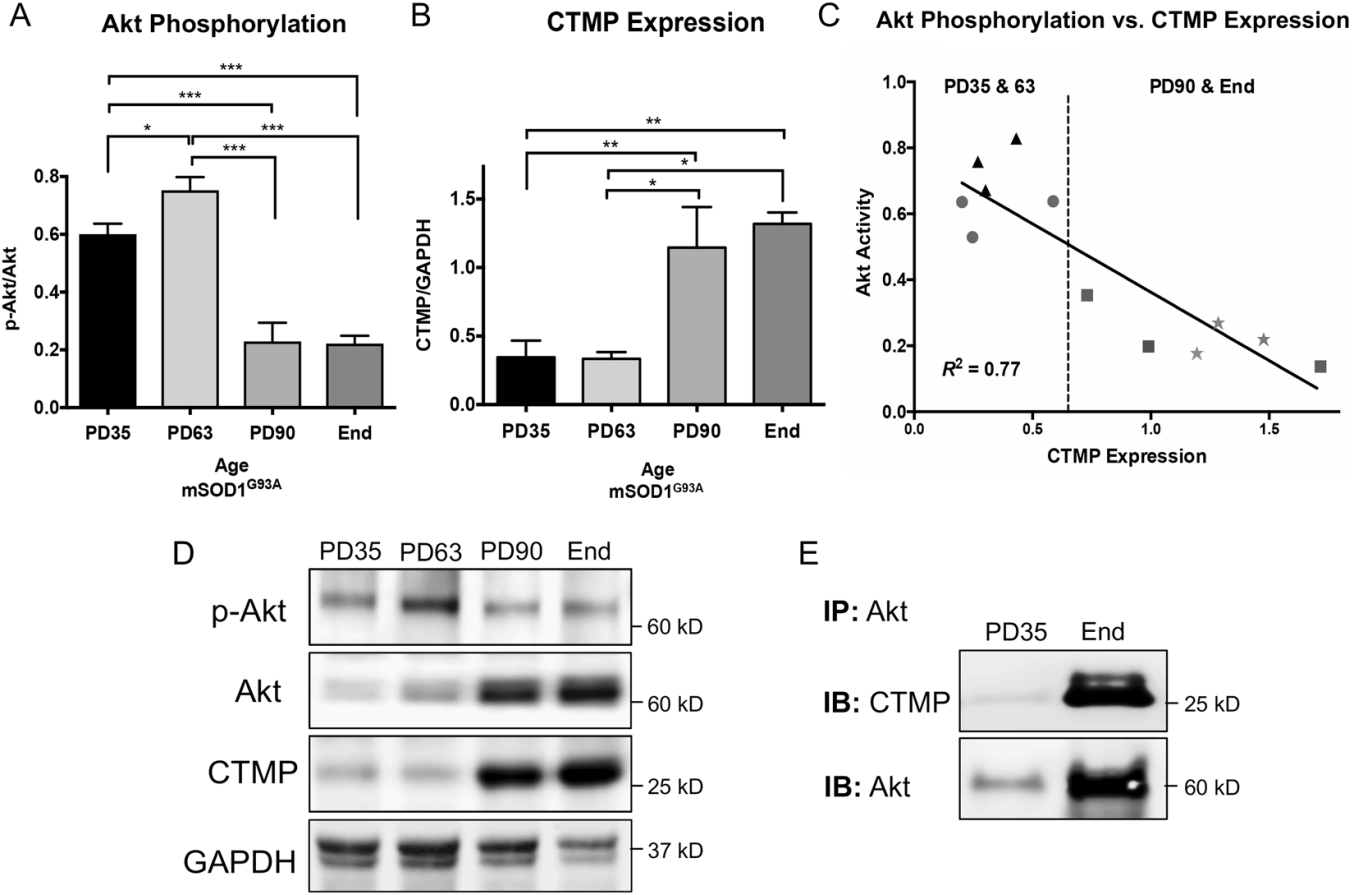
Changes in Akt signaling and CTMP expression, and their interaction during disease progression in mSOD1^G93A^ muscle. Akt phosphorylation significantly decreased by post-natal day (PD) 90 (**A**,**D**), while CTMP increased during this period (**B**,**D**). This relationship is inversely correlated throughout end-stage (~PD130) (**C**). CTMP exhibited considerable binding to Akt at end-stage compared to PD35, correlating with their respective expression levels at these time points, as shown via co-immunoprecipitation (**E**). This supports CTMP binding to Akt as a mechanism of Akt phosphorylation downregulation in mSOD1^G93A^ muscle. Blot images are cropped from different membranes used for data collection and analysis (See Supplemental Figures). *P < 0.05; **P < 0.01; ***P < 0.001.

To assess temporal changes in Akt phosphorylation in progressively denervated and atrophying mSOD1^G93A^ mouse skeletal muscle, gastrocnemius protein from post-natal day (PD)35 (pre-denervation), PD63 (pre-symptomatic), PD90 (near symptom onset) and end-stage was collected and phosphorylated and total Akt were assessed via Western blot (Fig. 1). As measured as a ratio of phosphorylated Akt to total Akt, Akt phosphorylation showed significant upregulation by PD63 compared to PD35 (*P* < 0.001) (Fig. 1A,D). Activity was then significantly reduced by PD90 compared to both PD35 and PD63 (both *P* < 0.001). At end-stage, Akt phosphorylation remained similar to the level observed at PD90 (Fig. 1A,D).

**Figure 2:**
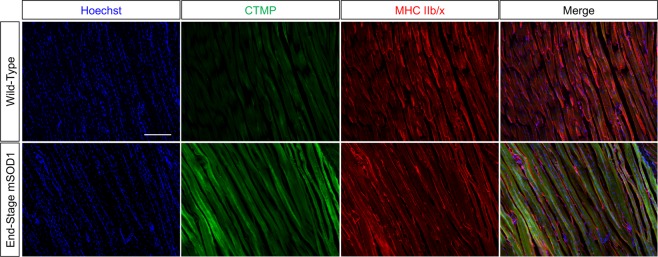
Immunofluorescence labeling of CTMP supports dramatic end-stage expression differences in WT and mSOD1^G93A^ gastrocnemius muscle. This image represents a longitudinal section through WT and end-stage mSOD1^G93A^ mouse gastrocnemius muscle. A notable increase in CTMP expression (green) observed in end-stage muscle tissue compared to muscle from WT mice. Red represents MHC IIb/x immunostaining for muscle fibers, and blue is Hoechst 33342 labeling of cell nuclei. Scale bar = 100 μm.

CTMP expression in this muscle tissue exhibited a near opposite expression pattern over time compared to Akt phosphorylation. Though CTMP expression remained unchanged between PD35 and PD63, its expression significantly increased by PD90 compared to both PD35 (*P* < 0.01) and PD63 (*P* < 0.05) (Fig. 1B,D). CTMP expression remained significantly elevated at end-stage compared to these earlier timepoints (*P* < 0.05), though its expression level was similar to that observed at PD90 (Fig. 1B,D). Regression analysis showed a very high inverse linear correlation between Akt phosphorylation and CTMP expression over time (*R*^2^ = −0.77) (Fig. 1C). Immunoprecipitation of Akt in pre-symptomatic mSOD1^G93A^ muscle and end-stage atrophic muscle from this mouse model showed binding of CTMP to Akt (Fig. 1E), corresponding to the increased expression of these proteins over time. This provides evidence that the correlation of CTMP increase and Akt phosphorylation may be due to CTMP binding-induced inhibition. In comparison to wild-type mouse gastrocnemius tissue, CTMP was qualitatively upregulated in end-stage mSOD1^G93A^ as observed via immunofluorescence labeling (Fig. 2).

### Inflammation and degradation pathways are upregulated in atrophying mSOD1^G93A^ muscle

**Figure 3:**
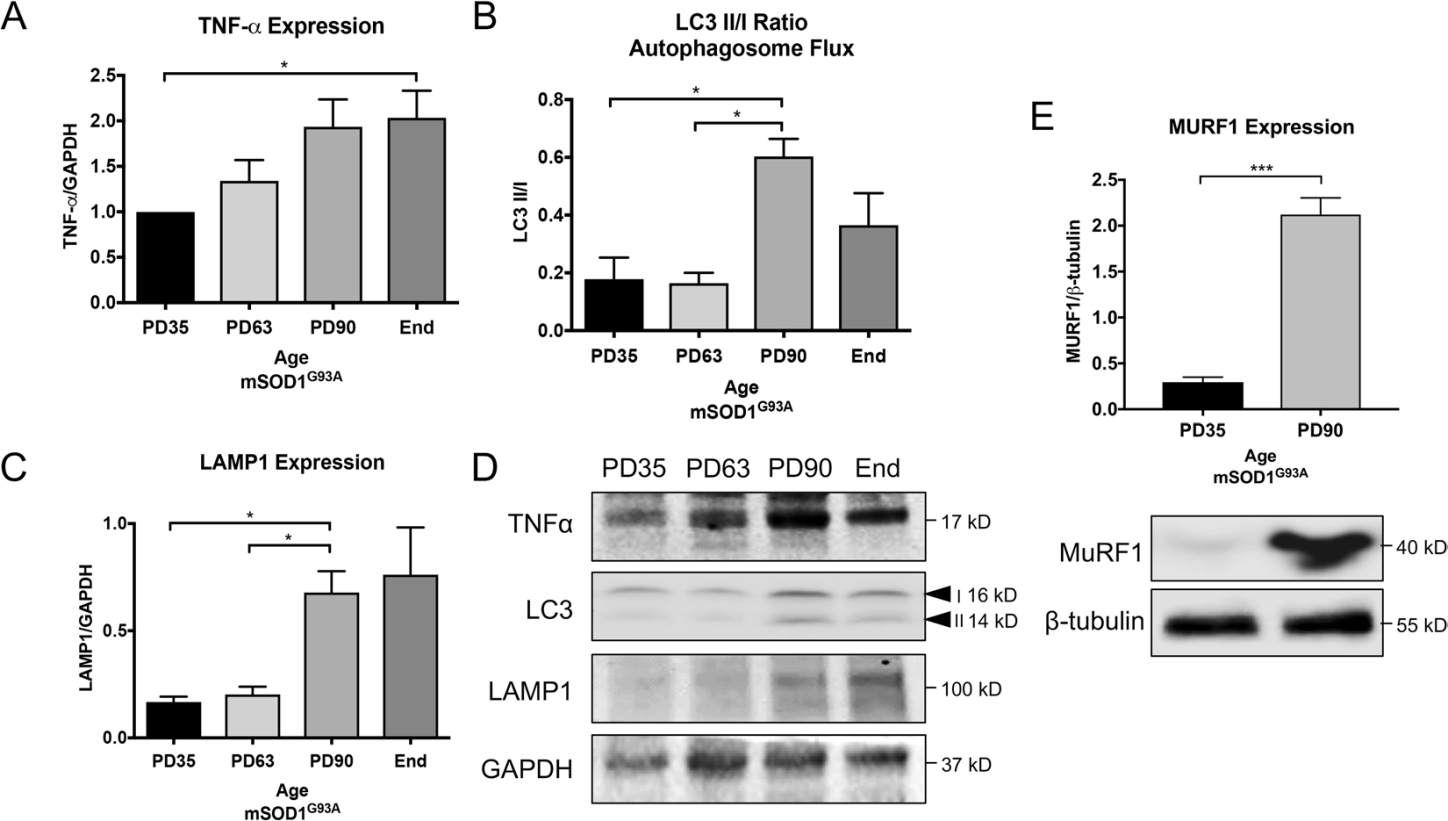
Inflammation, cell degradation markers and atrophy increase over time in mSOD1^G93A^ hindlimb muscle. TNFα increases over time in mSOD1^G93A^ gastrocnemius muscle tissue (**A**,**D**), as well as autophagosome (**B**,**D**) and lysosomal aggregation (**C**,**D**). The E3 ubiquitin ligase, MuRF-1 is significantly increased at PD90 compared to PD35, indicating increased muscle breakdown and atrophy as the disease progresses (**E**). Blot images are cropped from different membranes used for data collection and analysis (See Supplemental Figures). *P < 0.05.

Inflammation is a common characteristic of progressive muscle atrophy^25–27^, and E3 ubiquitin ligase MuRF-1 protein is known to be increased in late-stage mSOD1^G93A^ skeletal muscle^11^ and related to inflammation-induced muscle atrophy^28^. We found that TNFα levels gradually increased and were significantly higher at end-stage than early in the disease (PD35, *P* < 0.05, Fig. 3A,D). In looking at downstream cell degradation pathways associated with skeletal muscle break-down and Akt signaling, autophagosome flux, as indicated by the expression ratio of LC3 II to I, was increased by PD90 compared to earlier pre-symptomatic timepoints (*P* < 0.05, Fig. 3B,D). In addition, lysosome increase, as indicated through LAMP1 expression, was also significantly increased at PD90 compared to PD63 (*P* < 0.05) and PD35 (*P* < 0.05, Fig. 3C,D). Regarding muscle atrophy, our findings confirmed a significant upregulation of MuRF-1 over time in mSOD1^G93A^ gastrocnemius muscle (*P* < 0.05, Fig. 3E).

### An *in vitro* model of muscle atrophy shows increased CTMP expression and reduced Akt phosphorylation

**Figure 4:**
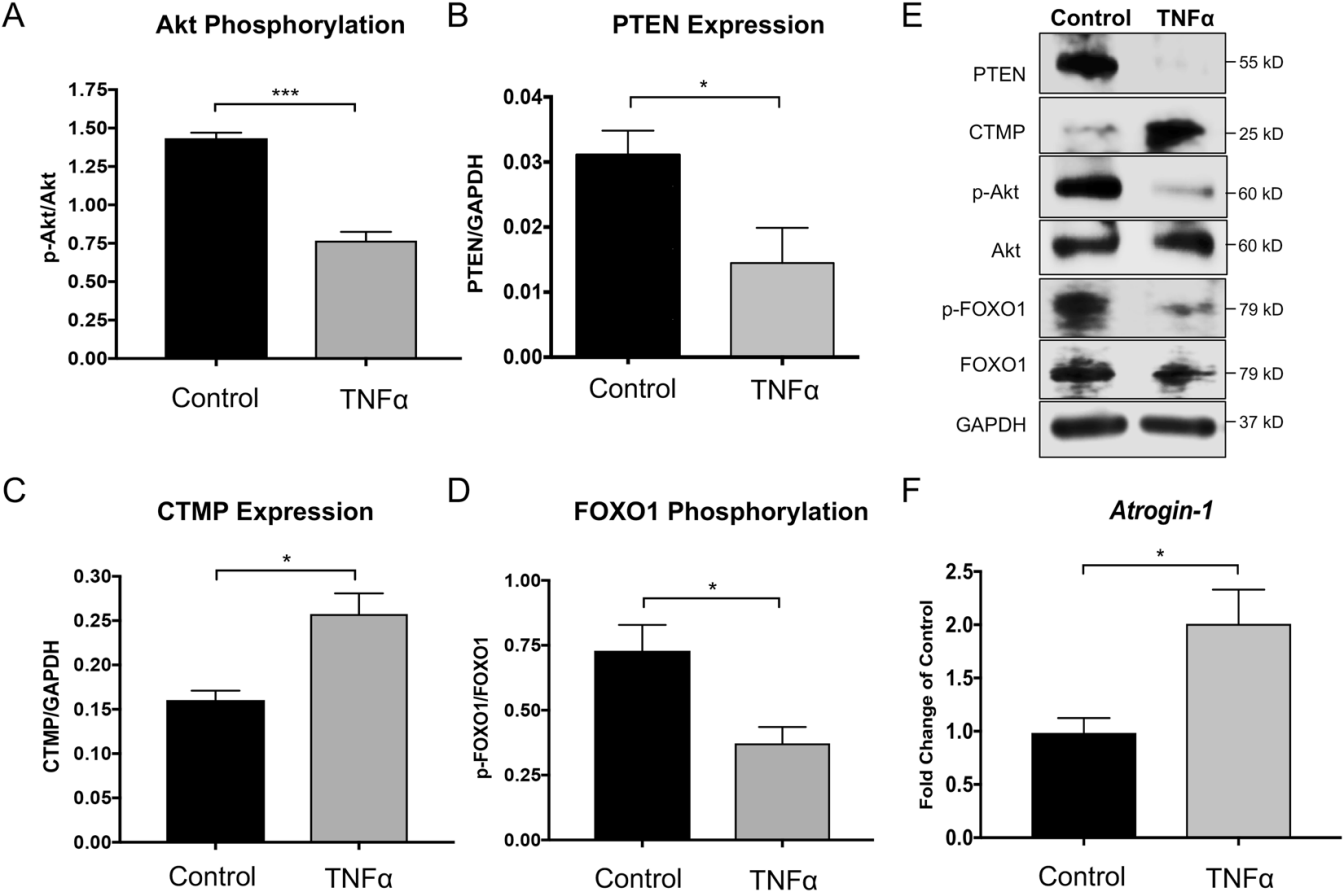
Treating differentiated murine C2C12 myotubes with 100 ng/mL TNFα for 24 hrs induced similar intracellular signaling pathway changes as observed in late-stage mSOD1^G93A^ mouse muscle tissue. Akt phosphorylation decreased following TNFα treatment (**A**,**E**). Meanwhile, PTEN expression was downregulated, suggesting it may not be a key modulator of Akt phosphorylation (**B**,**E**). However, CTMP expression significantly increased (**C**,**E**), as seen in the ALS mouse muscle. FOXO1 phosphorylation decreased (**D**,**E**), allowing it to permit transcription of atrophy genes such as *Atrogin-1*, which was significantly upregulated (**F**). Blot images are cropped from different membranes used for data collection and analysis (See Supplemental Figures). *P < 0.01; ***P < 0.001.

To investigate whether CTMP and Akt signaling were influenced in a similar way observed in late-stage mSOD1^G93A^ mouse gastrocnemius, we performed Western blot analysis. We found that Akt phosphorylation was significantly reduced 24 hours following TNFα treatment in differentiated C2C12 myotubes (*P* < 0.05, Fig. 4A,E). The expression of PTEN, a known suppressor of Akt activity, was downregulated in these cells (*P* < 0.05, Fig. 4B,E), suggesting it was not a likely factor in Akt phosphorylation reduction. Meanwhile, CTMP expression was significantly increased (*P* < 0.05, Fig. 4C,E), as observed in late-stage ALS mouse muscle. Reduced Akt signaling causes reduced FOXO1 phosphorylation, which enables its function of entering the nucleus to promote atrophy gene expression. Accordingly, we found FOXO1 phosphorylation was significantly decreased (*P* < 0.05, Fig. 4D,E). RT-PCR confirmed upregulation of *Atrogin-1* gene expression (*P* < 0.05, Fig. 4F), further corroborating our *in vivo* findings.

### CTMP knock-down rescues Akt phosphorylation *in vitro*

**Figure 5:**
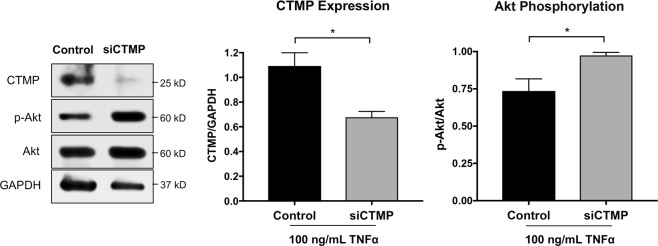
Knocking down CTMP in TNFα-treated differentiated C2C12 myotubes using siRNA rescued the decrease in Akt phosphorylation. Incubating differentiated TNFα-treated C2C12 myotubes with siRNA against CTMP for 73 hours significantly decreased CTMP expression and increased Akt phosphorylation, indicating a direct relationship between CTMP expression and Akt phosphorylation in this muscle atrophy model. Blot images are cropped from different membranes used for data collection and analysis (See Supplemental Figures). *P < 0.05.

Using the TNFα treated C2C12 cell model of muscle atrophy, we then tested whether knock-down of CTMP could increase Akt phosphorylation and confirm a direct relationship between CTMP and Akt signaling in atrophic muscle. After knocking down CTMP expression with CTMP-targeted siRNA, the significant decrease in CTMP expression (*P* < 0.05) corresponded with a significant increase of Akt phosphorylation compared with non-transfected control cells (*P* < 0.05) (Fig. 5).

## Discussion

This study is the first to show a direct connection between the expression of CTMP and downregulation of Akt phosphorylation in atrophic muscle. Importantly, this is also the first work to provide a potential mechanism for the downregulation of Akt activity in late-stage ALS mouse muscle, portraying it as a potential target. A neurocentric focus of ALS has long guided research and therapeutic investigation, though systemic influences have gained increasing attention as major contributors to onset and progression of the disease. Of these, target skeletal muscle, which is directly linked to motor dysfunction and paralysis, has been one of the most widely studied tissues. Much evidence suggests at least in mSOD1^G93A^ mutation-associated ALS, many pathologic changes in target skeletal muscle occur before symptom onset and motor neuron loss. In the mSOD1^G93A^ mouse model, a decrease in hindlimb muscle fiber size is noticeable between postnatal weeks eight & nine^29^, which follows closely after initial significant muscle denervation in this muscle group^5,6^. Early neuromuscular denervation triggers compensatory sprouting of motor axons, and ultimately, progresses toward disconnection from skeletal muscle. Several studies have investigated the cellular mechanisms that are characteristic of denervated skeletal muscle, particularly with regard to metabolic and cellular degradation pathways linked to progressive muscle atrophy^30,31^. As a primary pro-survival and key cell metabolism signaling kinase, reduced Akt phosphorylation and activity have been documented in symptomatic human and rodent ALS model skeletal muscle. We demonstrate here that skeletal muscle Akt phosphorylation significantly decreases over time in the mSOD1^G93A^ mouse model, with relatively increased levels at pre-symptomatic stages and significant downregulation approaching symptom onset.

Sustaining muscle mass depends heavily on innervation and neural stimulation of NMJs. As mentioned, hindlimb skeletal muscle is significantly denervated by post-natal day 47 in the mSOD1^G93A^ mouse^5,6^, and our findings in the present study indicate that Akt signaling and cell degradation-associated processes including autophagosome and lysosome aggregation and increased muscle atrophy are low during and through this period. However, these processes, as indicated by LC3 lipidation, LAMP-1 and MuRF-1 expression, respectively, significantly increase at later stages of disease when Akt signaling is greatly decreased. Others have observed increased Akt expression, as well as elevated Akt phosphorylation in skeletal muscle of mSOD1^G93A^ mice beginning in a pre-symptomatic stage of disease corresponding to our PD63 time point and continuing until end-stage^32^. However, this research group still observed muscle atrophy over the lifespan of the mice. One difference in their study and the present report is that they utilized a pool of different hindlimb muscles for protein analysis of Akt expression and phosphorylation, while we used only the medial gastrocnemius muscle. Different muscles are denervated at different rates in the mSOD1^G93A^ mouse based on the fiber-type content of a given muscle^33^. Motor axons with increased metabolic burden are the first to disconnect from NMJs^5^, and muscle fibers shown through electromyography to exhibit early motor dysfunction representative of denervation are fast twitch muscle fibers^33^. Medial gastrocnemius contains fast and slow twitch muscle fibers, and the fast twitch fibers are the first to undergo denervation, while some slower action muscle fibers maintain innervation^33^. Therefore, the use of pooled hindlimb muscles may include a disproportionate number of fast versus slow fibers, and may have different intracellular functions based on state of innervation leading to the increased Akt and its phosphorylation as the disease progresses in the mSOD1^G93A^ mouse^32^.

A detailed study showed that atrophy of the gastrocnemius muscle is observed shortly after the documented initial denervation period of 40–50 days of age in the mSOD^G93A^ mouse, and that it corresponds with onset of muscular dysfunction^34^. This evidence suggests, at least in the case of the medial gastrocnemius, that pre-symptomatic denervation may trigger early muscle atrophy. Interestingly, following post-natal day 47, compensatory collateral sprouting of motor axons to reinnervate disconnected NMJs occurs, and mice show no outward physical symptoms until closer to 100 days of age, though some minor motor deficits occur as early as post-natal day 55^34^. Still, this compensatory reinnervation may stimulate muscle metabolism and increased Akt signaling in middle pre-symptomatic stages of disease. This could explain why we observed significantly increased Akt phosphorylation at post-natal day 63 compared to day 35. Unfortunately, this sprouting and reinnervation diminishes over time and eventually a threshold level of NMJ denervation occurs, corresponding to reduced motor neuron numbers and contributing to progressive muscle dysfunction and increased muscle atrophy^35^. As shown here, by 90 days of age, the mice exhibit significantly decreased levels of Akt phosphorylation compared to day 63, and this low level is sustained through end-stage. One mechanism of NMJ denervation-mediated influence on muscle atrophy involves feedback inhibition of insulin signaling and subsequent downregulation of Akt phosphorylation^36^. Through this mechanism,  mammalian target of rapamycin (mTOR) and downstream S6 kinase activity feedback and inhibit this signaling pathway, which promotes upregulation of FOXO nuclear translocation and increased atrophy gene transcription. This is counterintuitive to the common understanding of the regulation of such processes by Akt and downstream signaling through mTOR^37–39^. However, this mechanism is sensible in the temporal context of muscle atrophy observed in ALS, with humans and rodents exhibiting reduced muscle function and significant muscle atrophy as the disease progresses^29,40,41^.

Although most studies concerning catabolic mechanisms contributing to muscle wasting in ALS have focused downstream of Akt, few studies have investigated the potential intracellular causes of Akt activity downregulation. We found a linear relationship between increased CTMP expression and reduced Akt phosphorylation in progressively atrophying mSOD1^G93A^ muscle. Since CTMP-mediated inhibition of Akt is dependent on its binding to Akt and preventing it from targeting the plasma membrane^20^, we immunoprecipitated Akt and found that it bound to CTMP  at a low level at the early-pre-symptomatic timepoint of PD35, indicative of its low expression at this point in time as shown in Fig. 1. However, at end-stage, when CTMP expression was significantly elevated, its binding to immunoprecipitated Akt was markedly increased. This is the first evidence showing a temporal relationship between Akt and CTMP, and confirming their interaction to potentially explain the linear inverse relationship between CTMP expression and Akt phosphorylation in progressively atrophying mSOD1^G93A^ muscle.

To more clearly assess the mechanisms associated with muscle atrophy, we utilized an established TNFα-induced C2C12 myotube atrophy model and examined Akt phosphorylation, CTMP expression, and related signaling processes. As reported by Wang *et al*.^42^, TNFα caused a significant downregulation of Akt phosphorylation in these cells. As anticipated, we also observed a subsequent downregulation of FOXO-1 phosphorylation associated with increased *Atrogin-1* expression. As seen in atrophic muscle in our ALS mouse model, CTMP expression also increased, which has not been reported. This increase in CTMP concomitant with downregulated Akt phosphorylation suggested the two could be associated. To examine whether PTEN could have been involved in the downregulation of Akt phosphorylation, we examined PTEN expression through Western blot analysis. Surprisingly, PTEN expression significantly decreased after TNFα treatment in the differentiated C2C12 cells, suggesting it was not likely responsible for Akt phosphorylation downregulation. Likewise, it further supported our hypothesis that CTMP played a role in reduced activity of Akt.

To confirm whether CTMP significantly influenced Akt phosphorylation in our *in vitro* model of muscle atrophy, knock-down of CTMP with specific siRNA resulted in an expected significant decrease in CTMP expression, but also significantly increased Akt phosphorylation. To our knowledge, this is the first evidence of CTMP modulating Akt phosphorylation in skeletal muscle atrophy. As Akt has been so widely studied in muscle atrophy in various conditions and diseases, this raises the question as to whether CTMP could be a common negative regulator of Akt activity and its downstream signaling, contributing to catabolic and atrophy processes in skeletal muscle. Not only is CTMP a potential target for therapy in neurologic injury, but also in skeletal muscle atrophy. Though we did not see increased CTMP upregulation early in the disease process in ALS mouse model muscle tissue, its expression pattern was similar to the observed increase in MuRF-1 expression between PD35 and PD90. As such, CTMP may still be exerting its negative influence on Akt activity leading to muscle atrophy, with its increased expression exacerbating the process over time.

Despite our results, the present study has some limitations and notable issues. First, the transition between PD63 and PD90 appears to be a critical period in the shift in metabolic processes and associated cell signaling. Future research examining skeletal muscle in the mSOD1^G93A^ mouse model at individual points during this time period could reveal interesting and important details concerning cell signaling involvement in the disease process. Though hindlimb muscle denervation significantly decreases several weeks earlier than symptom onset in the hindlimb muscles of this mouse model, transient compensatory sprouting occurs allowing for continued normal outward function of the muscle in the locomotion and ambulatory activities of the mouse^43–45^, as symptoms are not typically observed until near 100 days of age^46^. Also, the comparison of the effects of triggered muscle atrophy processes in a muscle cell line is not as optimal as proof from primary cell culture. Additional research into the biochemical processes primary skeletal muscle cells from mSOD1^G93A^ and WT control mice will allow for a more accurate perspective on the mechanistic role of CTMP in skeletal muscle cells under normal and atrophic conditions. Lastly, a correlation between our findings in the mSOD1^G93A^ mouse with human ALS patient muscle tissue would help anticipate the translational potential for any developed therapies.

**Figure 6:**
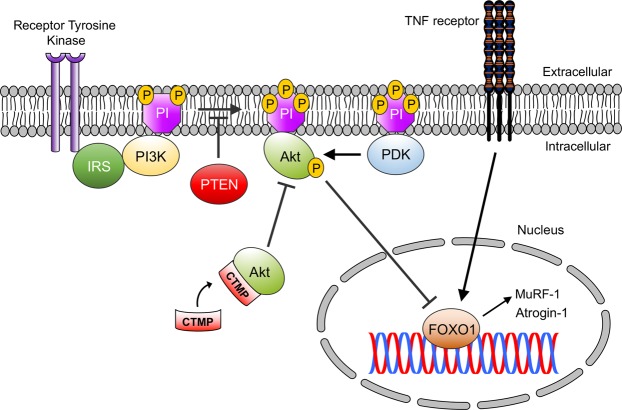
Schematic diagram for the proposed mechanism of CTMP-mediated Akt activity decrease and downstream signaling modulation in progressive muscle atrophy. The present study provides evidence that increased CTMP in atrophying mSOD1^G93A^ skeletal muscle correlates with Akt inactivation. The regulation of Akt signaling by CTMP may be due to binding of Akt and preventing its activation at the plasma membrane, leading to downstream increased expression in pro-atrophy genes and proteins, including Atrogin-1 and MuRF-1.

In conclusion, this study sheds new light on the regulation of Akt phosphorylation in mSOD1^G93A^ mouse muscle by CTMP and provides evidence that it plays a direct role in the consistently observed reduction in the activity of Akt and its downstream biochemical processes in atrophic muscle (Fig. 6). There are several reports of a negative role for CTMP in neuronal survival via reduced Akt signaling in neurological research, and this study suggests it could be a potential target in one of the earliest pathologies documented in ALS. Future research will more clearly elucidate the role of CTMP and the effects of limiting its inhibitory influence on Akt in ALS and other neuromuscular disorders.

## Methods

### Mouse muscle collection and biochemical preparation

Wild-type (WT) and 35d, 63d, 90d B6SJL mSOD1^G93A^ mice (Jackson Mice, Bar Harbor, ME) (n = 3–4 per group) were sacrificed via ketamine/xylazine and followed by rapid removal and flash freezing of gastrocnemius muscle tissue. To isolate protein, the muscle was minced and placed in radioimmunoprecipitation assay (RIPA) buffer with Halt protease and phosphatase inhibitor cocktail (Thermo Pierce). The minced muscle tissue in lysis buffer was then homogenized and left on ice for 30 min for additional lysis. Next, the lysed muscle tissue tubes were centrifuged at 13,000 rpm for 10 minutes to pellet non-digested debris and tissue. The supernatant was carefully removed, and concentrations of each sample was determined using a bicinchoninic acid (BCA) assay. All animal procedures were approved by the Institutional Animal Care and Use Committee of the Indiana University School of Dentistry and followed the National Institutes of Health (NIH) Guidelines on the humane care and use of laboratory animals.

### C2C12 myoblast culture, differentiation and CTMP knock-down

To test whether CTMP knock-down affects Akt phosphorylation in an *in vitro* model of muscle atrophy, C2C12 myoblasts (ATCC) were cultured in 6 well plates at a density of 4 × 10^5^ cells/well for 24 hrs in normal growth medium (Dulbecco’s Modified Eagle Medium [DMEM] supplemented with 10% fetal bovine serum [FBS] and 1% penicillin/streptomycin [P/S], Life Technologies, Inc. Carlsbad, CA). Upon reaching 90% confluency, the normal growth medium was removed, and cells were treated with 50 nM CTMP siRNA or 50 nM scrambled non-targeting siRNA in proprietary siRNA Transfection Medium (Santa Cruz Biotechnologies) for 5 hours. Then, 1 mL normal growth medium was added for an additional 24 hours. The next day, normal growth medium was removed, the cells were washed in DMEM and then incubated in differentiation medium (DMEM + 2% FBS + 1% P/S) for 72 hours. After this period, myotubes were prominent indicating differentiation, and 100 ng/mL TNFα was added in DMEM for 24 hrs to induce an atrophic state as described by Wang *et al*.^42^. Whole cell lysates of each sample were then prepared in 1x Laemmli buffer (Bio-Rad) with 5% β-mercaptoethanol for Western blot analysis. Experiments were repeated in triplicate.

### Western blot

Western blot data was performed as described previously^47^. In brief, 20–30 μg protein was loaded onto 4–20% gradient tris-glycine gels (TGX, Bio-Rad) and the protein was transferred onto polyvinylidinefluoride (PVDF) membranes via a TransBlot machine (Bio-Rad). Following a wash in phosphate buffered saline (PBS), the membranes were blocked in milk blocking buffer (ThermoPierce) for 1 hour at room temperature. Then, the membranes were incubated with the following primary antibodies in PBS + 0.1% Tween 20 (PBST) + milk blocking buffer (ThermoPierce) overnight at 4 °C or 1 hr at room temperature: mouse anti-pan Akt, rabbit anti-p-Akt^Ser473^, rabbit anti-LAMP1 (1:1,000), a marker for lysosomal activation, TNFα (1:1,000), rabbit anti-CTMP (1:1,000) (Cell Signaling Inc.), rabbit anti-LC3 (1:500; Abcam), a marker of autophagosome flux, rabbit anti-MURF-1, a marker of atrophy (1:1000, EMC Biosciences), and mouse anti-β-tubulin (Cell Signaling Inc.) and rabbit anti-glyceraldehyde 3-phosphate dehydrogenase (GAPDH) (Sigma-Aldrich) were used as loading controls. Following primary antibody incubation, the membranes were washed 3x with PBST and incubated with either Li-Cor IRdye 600 or 800 for imaging on a Li-Cor Odyssey Fc machine, or horseradish peroxidase (HRP)-conjugated secondary antibodies for use in enhanced chemiluminescence (ECL) imaging using a Li-Cor c-DiGit imaging system (Li-Cor). After secondary antibody incubation and washes with PBST, IRDye-incubated membranes were immediately imaged. Those incubated with HRP-conjugated secondary antibodies were exposed to ECL reagents (ThermoPierce ECL Kit, ThermoPierce; Western Sure Premium ECL, Li-Cor) for 1–5 min. Excess ECL reagent was blotted off of the membranes and they were imaged as described above. Resulting bands were converted to gray scale and densitometry analysis was performed using ImageJ software (NIH).

### Co-immunoprecipitation of Akt and CTMP

Co-immunoprecipitation of Akt total protein and bound CTMP from mSOD1^G93A^ gastrocnemius muscle followed previously published protocols with modification (Wu *et al*., 2017). In brief, Bio-Rad Rapid Magnetic Beads (Bio-Rad) were used to bind pan-Akt antibody (Cell Signaling, 1:50) and this complex was allowed to immunoreact with PD35 and end-stage mSOD1^G93A^ mouse gastrocnemius muscle protein lysate under rotation for 1 hr at room temperature. Non-antibody protein exposure (beads only) was used as a negative control. The bound protein was eluted for Western blotting via addition of 1x Laemmli sample buffer (Bio-Rad) and heating at 70 °C for 10 min. After heating, the sample buffer containing the immunoprecipitated and associated proteins was transferred to a separate tube for Western blot.

### Primers

Primers for real-time PCR were as follows (sense and antisense, 5′ to 3′): Atrogin-1/Mafbx: TGAAT AGCATCCAGATCAGCA, GATGTTCAGTTGTAAGCACACAG; GAPDH: AATGGTGAAGGTCGGTGTG, GTGGAGTCATACTGGAACATGTAG (PrimeTime qPCR Primers, IDT, Coralville, IA).

### RNA Isolation and Real-Time PCR

Total RNA was isolated from differentiated C2C12 cells and reverse transcription (RT) and qPCR were performed to assess atrophy gene expression (PureLink RNA Isolation Kit, Invitrogen, Carlsbad, CA). Total isolated RNA was reverse transcribed using a High-Capacity cDNA Reverse Transcription Kit (Applied Biosystems, Foster City, CA) and a Bio-Rad C1000 thermal cycler (Hercules, CA) according to the manufacturer’s instructions. Real-time PCR was performed in an ABI Prism 7000 system (Applied Biosystems) using the obtained complementary DNA and a Maxima SYBR Green/ROX qPCR kit (ThermoPierce). GAPDH was used as an internal control.

### CTMP Immunofluorescence Labeling

For muscle tissue collection and immunofluorescence labeling, previously published methods were used^6^. In brief, end-stage wild-type (WT) and mSOD1^G93A^ mice (n = 3/group) were sacrificed using ketamine/xylazine, and perfused with 0.1 M phosphate buffered saline (PBS) followed by 4% paraformaldehyde solution in PBS to fix the tissue. The medial gastrocnemius was dissected and placed in the same fixative for 30 minutes at 4 °C. The muscle tissue was then washed with PBS and placed in 30% sucrose in PBS for cryopreservation overnight at 4 °C. After cryopreservation, the muscle tissue was embedded in TissueTek OCT media and serially sectioned longitudinally at 20 μm using a cryostat (Leica) and mounted on SuperFrost Plus microscope slides (Fisher Scientific). Four muscle sections at 200 μm intervals were selected for labeling per mouse. For immunofluorescence labeling of the muscle for CTMP, the tissue was permeabilized in PBS + 0.1% Triton-X 100 (PBST, Fisher Scientific) and blocked for non-specific labeling using 10% normal goat serum in PBST (blocking buffer) for 1 hr at room temperature. Then, the slides were incubated in blocking buffer with rabbit anti-THEM4 (CTMP) antibody (1:100, Abcam) and combined muscle fiber type antibodies, mouse anti-myosin heavy chain IIb and IIx (MHC IIb/x) (1:50, Developmental Studies Hybridoma Bank, University of Iowa) for 1 hr at room temperature. Next, the slides were washed and incubated in AlexaFluor-conjugated goat anti-rabbit and goat anti-mouse secondary antibody (1:200, Jackson Immunoresearch) and Hoechst 33342, for labeling nuclei (5 μg/mL, Sigma-Aldrich) for 1 hr at room temperature. The sections were then washed and coverslipped in Anti-Fade Gold aqueous mounting media (Life Technologies, Inc.) and left to dry before imaging. Imaging was performed on a Nikon epifluorescent microscope.

### Statistical analysis

All values are presented as mean +/−SEM. Comparisons between three or more groups was performed using a one-way ANOVA with Newman-Keuls post-hoc analysis if significance was determined. Comparisons between two groups were made using an unpaired *t*-test. All statistical analysis and graphing were completed using GraphPad Prism 6.0 software (GraphPad, Inc). *p* < 0.05 was considered statistically significant.

## Supplementary information

Supplementary Information

## Data Availability

The datasets generated during and/or analyzed during the current study are available from the corresponding author on reasonable request.
