# Supplementary material for: Carboxyl-terminal modulator protein regulates Akt signaling during skeletal muscle atrophy in vitro and a mouse model of amyotrophic lateral sclerosis
Source: Sci Rep. 2019 Mar 8;9:3920. doi: 10.1038/s41598-019-40553-2 (PMC6408440; doi:10.1038/s41598-019-40553-2)
Supplement: Supplementary file 1 — Supplementary Information [file 41598_2019_40553_MOESM1_ESM.pdf]

Carboxyl-terminal modulator protein regulates Akt signaling during skeletal muscle atrophy *in vitro* and a mouse model of amyotrophic lateral sclerosis

Junmei Wang[1], Colin M. E. Fry[2], and Chandler L. Walker[1,2,3*]

[1]Department of Biomedical and Applied Sciences, Indiana University School of Dentistry, Indianapolis, IN 46202, USA
[2]Department of Anatomy and Cell Biology, Indiana University School of Medicine, Indianapolis, IN 46202, USA
[3]Neuromuscular Research Group, Richard L. Roudebush Veterans Affairs Medical Center, Indianapolis, IN 46202, USA

***Corresponding Author:**
Chandler L. Walker, Ph.D.
Email: chalwalk@iu.edu

Supplemental Figure 1. Raw Western Blots for Figure 1

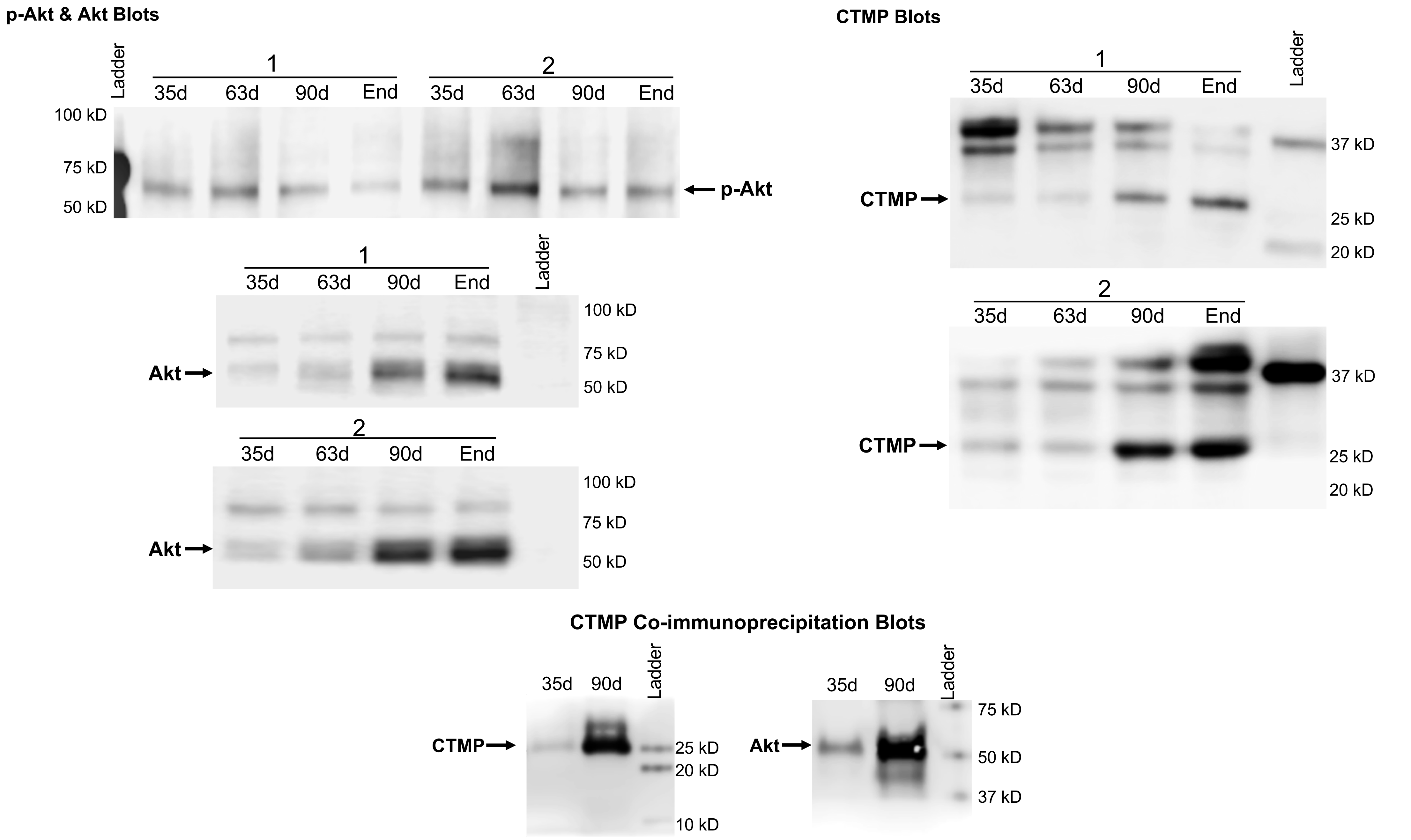

Supplemental Figure 2. Raw Western Blots for Figure 3

LAMP, GAPDH and LC3 Blot (Exposure 1)

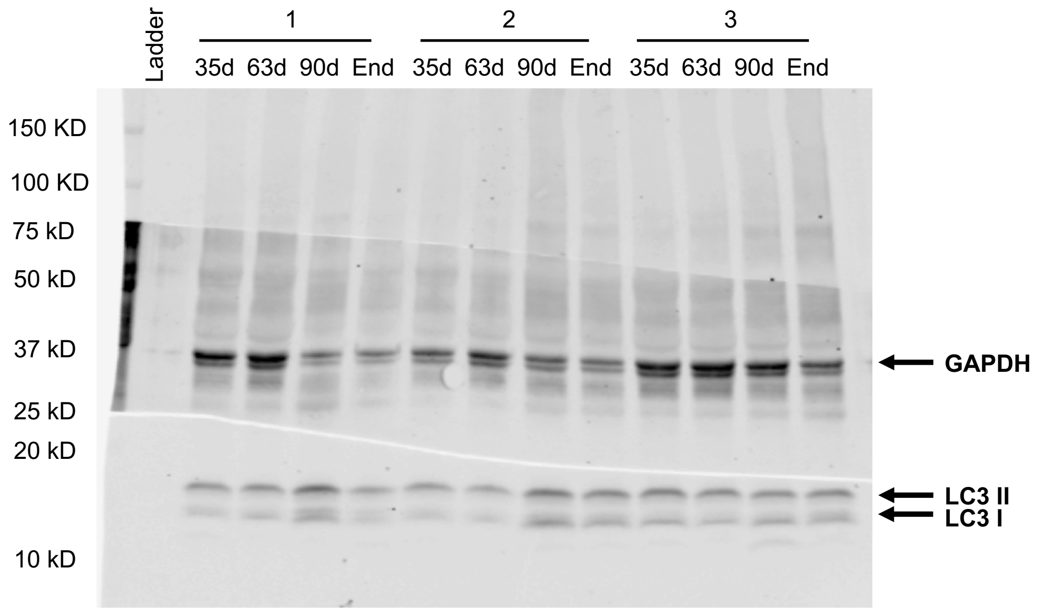

LAMP1, GAPDH and LC3 Blot (Exposure 2)

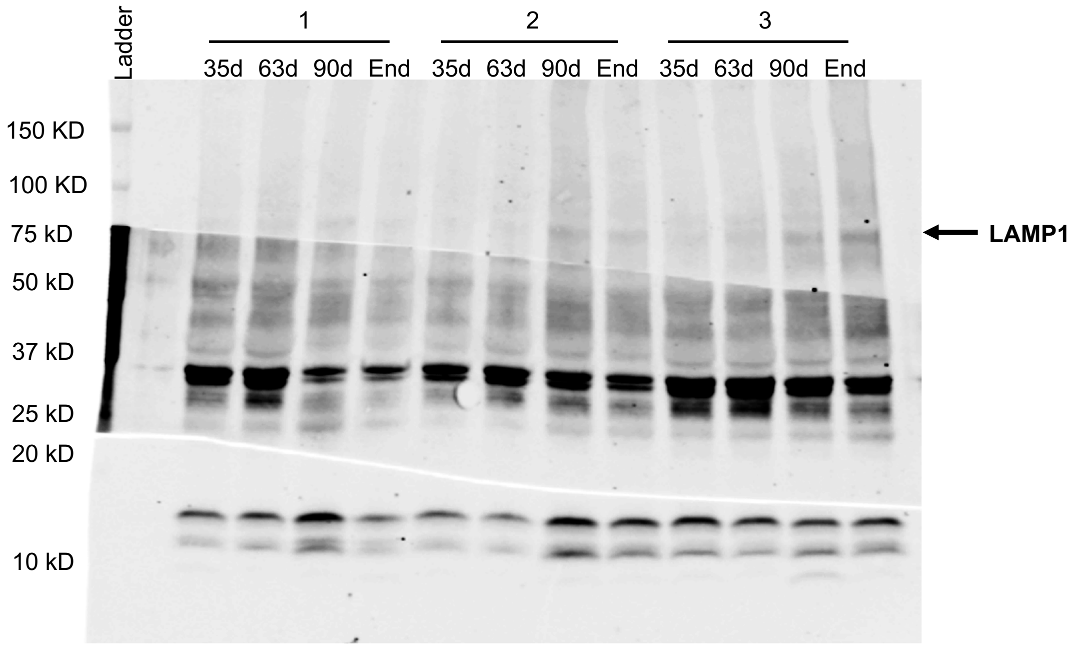

TNFα Blot

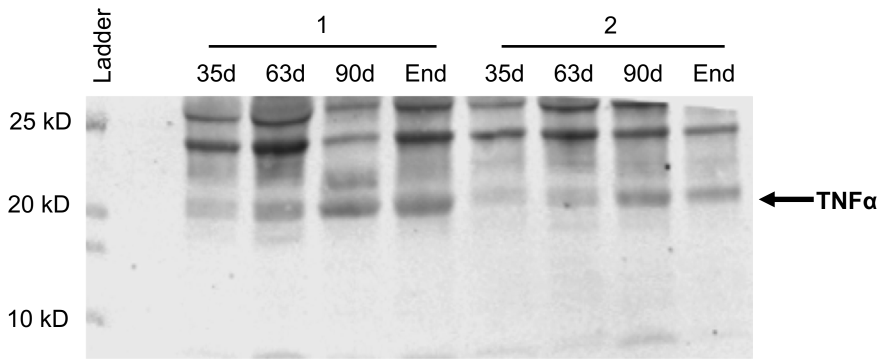

MurF1 Blot

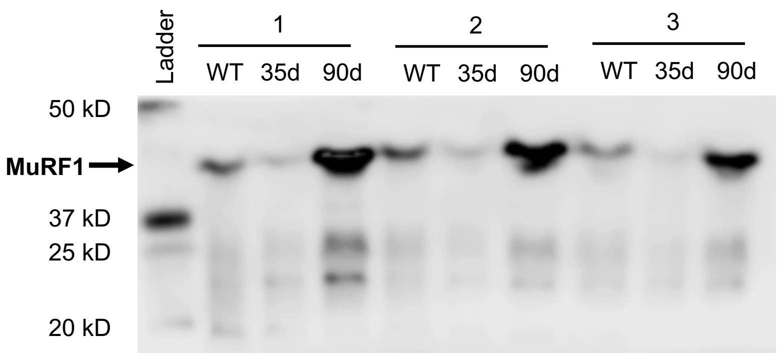

B-tubulin Blot

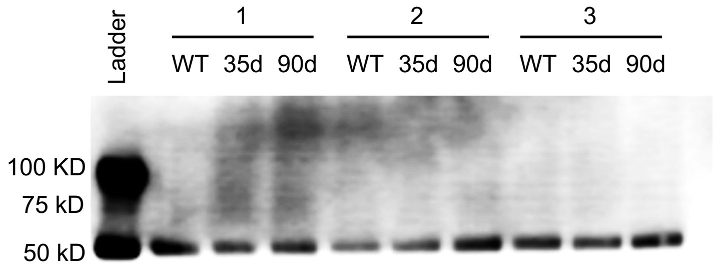

Supplemental Figure 3. Raw Western Blots for Figure 4

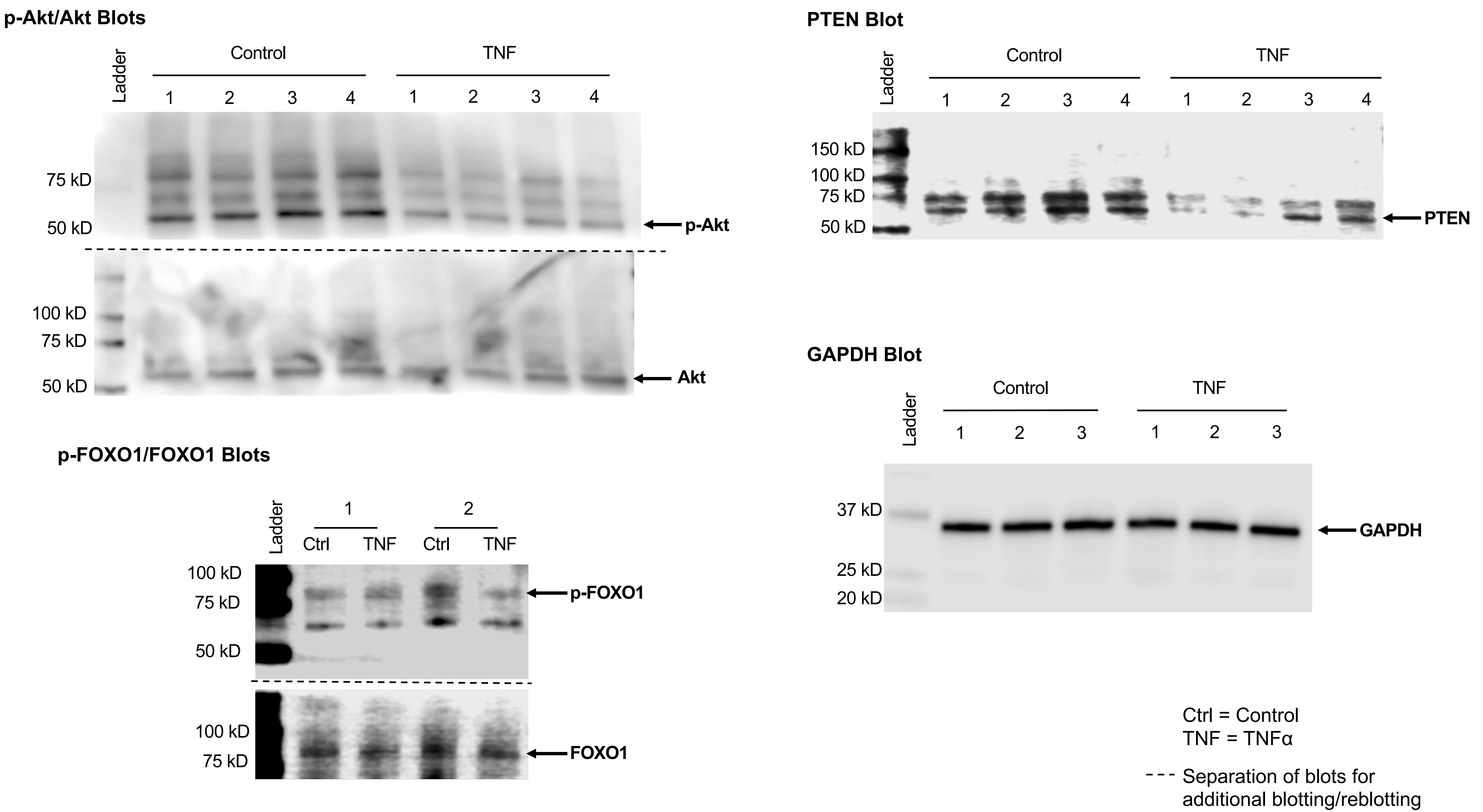

Supplemental Figure 4. Raw Western Blots for Figure 5

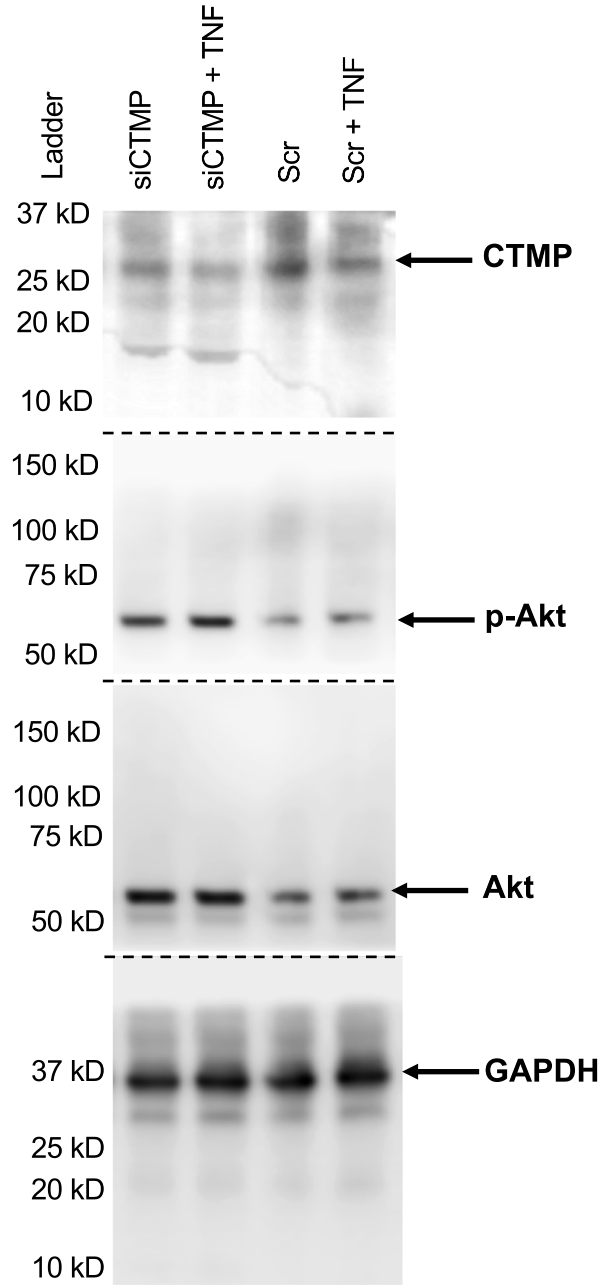

Scr = Scrambled siRNA
siCTMP = CTMP targeted siRNA
TNF = TNFα

--- Separation of blots for additional blotting/reblotting
